# Parasitic Disease Surveillance, Mississippi, USA

**DOI:** 10.3201/eid2708.204318

**Published:** 2021-08

**Authors:** Richard S. Bradbury, Meredith Lane, Irene Arguello, Sukwan Handali, Gretchen Cooley, Nils Pilotte, John M. Williams, Sam Jameson, Susan P. Montgomery, Kathryn Hellmann, Michelle Tharp, Lisa Haynie, Regina Galloway, Bruce Brackin, Brian Kirmse, Lisa Stempak, Paul Byers, Steven Williams, Fazlay Faruque, Charlotte V. Hobbs

**Affiliations:** Centers for Disease Control and Prevention, Atlanta, Georgia, USA (R.S. Bradbury, M. Lane, S. Handali, G. Cooley, S.P. Montgomery); Synergy America, Inc., Atlanta (M. Lane);; University of Mississippi Medical Center, Jackson, Mississippi, USA (I. Arguello, J.M. Williams, S. Jameson, K. Hellmann, M. Tharp, L. Haynie, R. Galloway, B. Kirmse, L. Stempak, F. Faruque, C.V. Hobbs);; Smith College, North Hampton, Massachusetts, USA (N. Pilotte, S. Williams);; Mississippi State Department of Health, Jackson (B. Brackin, P. Byers)

**Keywords:** Soil-transmitted helminths, *Strongyloides*, strongyloidiasis, *Toxocara*, toxocariasis, *Cryptosporidium*, cryptosporidiosis, giardiasis, parasites, pediatric, zoonoses, Mississippi, United States

## Abstract

Surveillance for soil-transmitted helminths, strongyloidiasis, cryptosporidiosis, and giardiasis was conducted in Mississippi, USA. PCR performed on 224 fecal samples for all soil-transmitted helminths and on 370 samples for only *Necator americanus* and *Strongyloides stercoralis* identified 1 *S. stercoralis* infection. Seroprevalences were 8.8% for *Toxocara*, 27.4% for *Cryptosporidium*, 5.7% for *Giardia*, and 0.2% for *Strongyloides* parasites.

Human populations in the state of Mississippi and the rest of the southeastern United States have historically been at risk for hookworm and other parasitic diseases (*1*,*2*). With improved sanitation and economic development, soil-transmitted helminths (STH), including the hookworms *Ascaris lumbricoides* and *Trichuris trichiura*, were presumed to have been eliminated. However, a recent report of continued hookworm and strongyloidiasis transmission in a community without access to proper sanitation in Alabama, USA, has challenged this assumption (*3*).

## The Study

To investigate the current prevalence of these infections, we conducted a pilot study to identify STH and other potentially endemic parasitic infections in convenience samples of specimens collected from patients in Mississippi. We deidentified fresh fecal samples submitted for diagnostic testing from patients at the University of Mississippi Medical Center (UMMC; Jackson, Mississippi, USA) during March 30, 2017–February 22, 2018, and serum samples submitted during October 28, 2017–March 29, 2018. This study was approved by the UMMC Institutional Review Board; the Centers for Disease Control and Prevention (CDC) was determined to be nonengaged and therefore did not undertake a separate institutional review board review.

We froze two 250-mg aliquots of feces for later DNA extraction. Where sample volume allowed, we performed microscopic examination using the saturated salt (specific gravity 1.2) passive flotation method as previously described (*4*). We extracted DNA by using the SurePrep Soil DNA isolation kit (ThermoFisher, https://www.thermofisher.com) after conducting initial bead beating for 3 minutes using zirconium beads. We stored DNA extracts at −80°C and sent them to the CDC for real-time PCR analysis. At CDC, each sample was initially tested for inhibition and poor DNA extraction by a real-time PCR assay targeting the human cytochrome B gene (*5*). Samples positive by this inhibition and extraction control were then tested by multiparallel real-time PCR for STH (*6*). A cycle threshold (C_t_) <35 was considered to represent a positive result. Any positive PCR results were confirmed by duplicate testing.

We froze the deidentified serum samples at –80°C and sent them to CDC, where they were tested for antibodies to *Toxocara* spp., *S. stercoralis*, *Cryptosporidium* spp., and *G. duodenalis* using MAGPIX multiplex serology (ThermoFisher) (Appendix) to detect evidence of prior exposure. For statistical calculations, we used Excel (Microsoft, https://www.microsoft.com) and R version 3.3.1 (https://www.r-project.org).

A total of 650 fecal samples were obtained from UMMC patients. The median age of patients providing fecal samples for this analysis was 56 years (range 2–95 years). We obtained samples sufficient to perform saturated salt centrifugal flotation on 507 samples (80%). We found no samples to contain helminth eggs or larvae. Sufficient sample for DNA extraction was available for 631 (99.5%) samples. Of these fecal DNA extracts, a negative inhibition and extraction control excluded 37 samples. We tested 224 DNA extracts for *Ancylostoma* spp., *N. americanus*, *S. stercoralis*, *A. lumbricoides*, and *T. trichiura* by real-time PCR. (Table 1)

**Table 1:** Results of microscopic examination and real-time PCR testing for soil-transmitted helminth and Strongyloides stercoralis infection on postdiagnostic fecal samples from patients at University of Mississippi Medical Center, Jackson, Mississippi, USA*

Method	Inhibition and extraction control	*S. stercoralis*	*Necator americanus*	*Ascaris lumbricoides*	*Trichuris trichiura*	*Ancylostoma* spp.	Other parasite species
Saturated salt centrifugal flotation	NA	0/507 (0)	0/507 (0)	0/507 (0)	0/507 (0)	0/507 (0)	0/507 (0)
Real-time PCR	594/631 (94.1)	1/594 (0.2)	0/594 (0)	0/224 (0)	0/224 (0)	0/224 (0)	NA
*Values are no. (%) unless indicated. NA, not applicable.							

Because prior work in Alabama (*3*) detected only *N. americanus* and *S. stercoralis* infections, we screened an additional 370 DNA extracts for these helminths only. Of these 370 samples, 2 DNA extracts yielded positive amplicons for *S. stercoralis* (C_t_ 29.57 and 30.48). The first of these samples (C_t_ 29.57) yielded no amplification curve on repeat testing and was interpreted as representing an initial false-positive result. The second sample (C_t_ 30.48) was positive upon confirmatory retesting (C_t_ 28.52 and 30.49). (Table 1)

**Figure 1:**
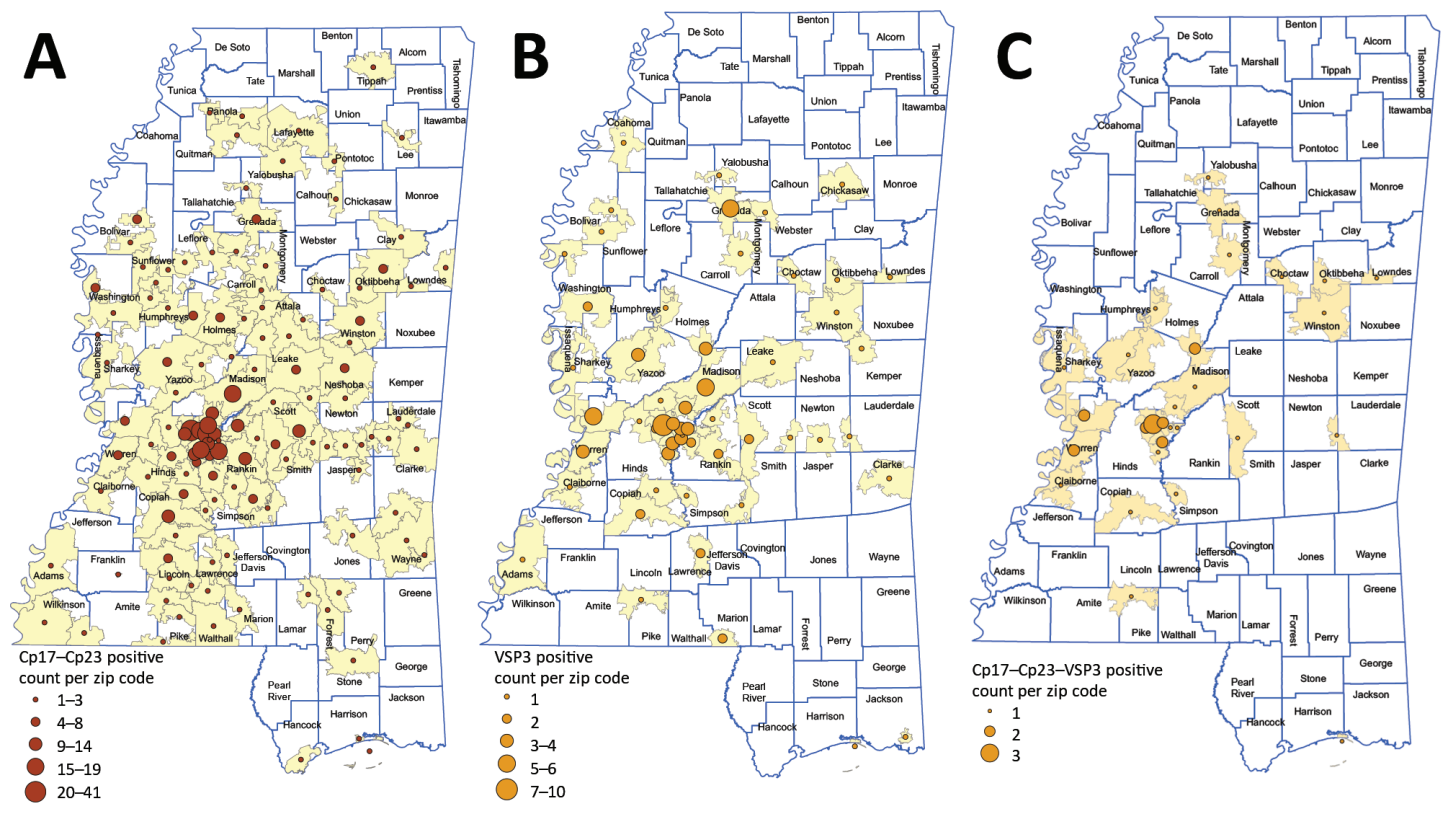
Places of residence of participants with antibody levels suggesting prior exposure to *Cryptosporidium* spp. Cp17 and Cp23 (n = 538) (A), *Giardia duodenalis* VSP3 (n = 111) (B), and *Cryptosporidium* spp. Cp17 and Cp23 and *Giardia duodenalis* VSP3 (combined) (n = 38) (C), Mississippi, USA. All serologic assays were performed using MAGPIX multiplex recombinant antigen beads (ThermoFisher, https://www.thermofisher.com) on convenience serum samples collected at the University of Mississippi Medical Center (Jackson, MS, USA) during October 28, 2017–March 29, 2018. Only those samples confirmed by a subsequent *S. stercoralis* crude L3 larval antigen (CrAg) ELISA are included.

**Figure 2:**
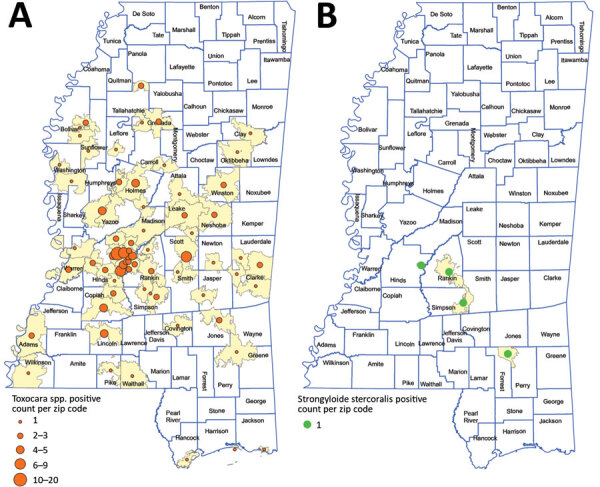
Places of residence of participants with antibody levels suggesting prior exposure to *Toxocara* spp. Tc-CTL-1 (n = 172) (A) and *Strongyloides stercoralis* Ss-NIE-1 (n = 4) (B) , Mississippi, USA. All serologic assays were performed using MAGPIX multiplex recombinant antigen beads (ThermoFisher, https://www.thermofisher.com) on convenience serum samples collected at the University of Mississippi Medical Center (Jackson, MS, USA) during October 28, 2017–March 29, 2018. Only those samples confirmed by a subsequent *S. stercoralis* crude L3 larval antigen (CrAg) ELISA are included.

A total of 1,960 postdiagnostic serum samples from Mississippi residents were available for multiplex serologic testing. The median age of patients providing serum samples for this analysis was 38 years (range 0–94 years). Of the 1,960 samples, 646 (33.0%) reacted with the Cp17 antigen of *C. parvum* (range 87–48,448 mean fluorescence intensity [MFI]), and 1,076 (54.9%) reacted with Cp23 (range 377–56,727 MFI). Of those samples, 538 (27.4%) reacted with both *C. parvum* antigens (Figure 1, panel A), suggesting prior *Cryptosporidium* species infection. A total of 111 samples (5.7%) reacted with the *G. duodenalis* VSP3 antigen (range 84–48,547 MFI) (Figure 1, panel B). A total of 38 (1.9%) samples contained antibodies to the Cp17, Cp23 and VSP3 antigens (Figure 1, panel C), demonstrating prior exposure to both *Cryptosporidium* and *G. duodenalis* infections. A total of 172 (8.8%) samples contained antibodies to *Toxocara* spp. Tc-CTL-1 antigen (range 23.2–33,814 MFI) (Table 2; Figure 2, panel A). When *Toxocara*-seropositive participants <6 years of age were excluded, 167/1,814 (9.2%) of UMMC patient samples were seropositive. A total of 9 (0.4%) samples contained antibodies reacting with the recombinant *S. stercoralis* NIE-1 antigen (range 16.2–11248 MFI) in MAGPIX serologic testing, of which 4 (0.2%) were positive in the confirmatory *S. stercoralis* CrAg-ELISA (range 9.94–57.7 IU/mL) (Figure 2, panel B).

**Table 2:** Results of multiplex serologic testing for antibodies suggesting prior exposure to *Toxocara* spp., *Giardia duodenalis*, and *Cryptosporidium* spp. on 1,960 postdiagnostic serum samples from patients at University of Mississippi Medical Center, Jackson, Mississippi, USA*

Parasite antigen used						
*Toxocara* spp. Tc-CTL-1	*S. stercoralis* rSs-NIE-1 plus CrAg-ELISA†	*G. duodenalis* VSP3	*C. parvum* Cp17	*C. parvum* Cp23	*C. parvum* Cp17 + Cp23‡	*C. parvum* Cp17 + Cp23 and *G. duodenalis* VSP3
172 (8.8)	4 (0.2)	111 (5.7)	646 (33.0)	1,076 (54.9)	538 (27.4)	38 (1.9)

*All values are no. (%). †8 samples were found to be positive by the rSs-NIE-1 MAGPIX multiplex serologic assay (ThermoFisher, https://www.thermofisher.com), but only 4 reacted in the confirmatory *S. stercoralis* crude L3 larval antigen (CrAg) ELISA. ‡Only samples reactive to both Cp17 and Cp23 were considered positive for *Cryptosporidium* spp. exposure.

## Conclusions

The results of this limited pilot study suggest a low prevalence of STH infections in Mississippi but that rare infections with *S. stercoralis* might be found in Mississippi residents. The single case confirmed by real-time PCR tests likely represents active infection. Because >80% of patients with strongyloidiasis serorevert within 18 months after successful treatment (*7*), the 4 confirmed antibody-positive serum samples also likely represent active cases of strongyloidiasis. No linked immigration or travel history data on patients providing these samples were available, so whether these infections were acquired within the United States is unknown. Combined with the recent finding of strongyloidiasis in a rural community from Alabama (*3*), these data should encourage more focused sampling of areas with poor sanitation and hygiene, high levels of poverty, and poor access to healthcare for potential residual foci of endemic STH and strongyloidiasis transmission in Mississippi and the wider southeastern United States.

The total *Toxocara* spp. seroprevalence in all participants in this study was 8.8%, which is higher than the average prevalence reported by the most recent National Health and Nutrition Examination Survey study (*8*). Although these results are not directly comparable because of different sampling methods, the potentially high *Toxocara* spp. seroprevalence in Mississippi warrants further investigation.

The seroprevalence results of this study suggest that prior exposure to *Cryptosporidium* spp. is common in Mississippi. Only 5.7% of the postdiagnostic serum samples were found to have serologic evidence of prior exposure to *G. duodenalis* infection. A small number of samples (1.9%) contained antibodies reacting with the 3 antigens Cp17, Cp23, and VSP3, indicating prior exposure to *Cryptosporidium* spp. and *G. duodenalis* infection. Further investigation of the epidemiology of waterborne protozoan infection in Mississippi, including determination of the actual prevalence and distribution using systematic sampling and determination of the species and subtypes infecting persons, is warranted.

The absence of any positive findings by microscopic examination or PCR for the STH suggests that such infections are uncommon in the general Mississippi population. We found high seroprevalence of antibodies to *Toxocara* spp. in Mississippi. Although this finding could indicate increased exposure to this infectious agent compared with the national average, our data do not enable determination of the sources of increased infection or overall annual incidence of disease. Further studies on the epidemiology and prevalence of parasitic diseases in the state of Mississippi are indicated.

In conclusion, this convenience sampling study did not find evidence of high STH prevalence in Mississippi. However, we did identify several likely current cases of strongyloidiasis and relatively high rates of *Toxocara* exposure. We recommend further investigation with larger sample sizes to more clearly define the true extent of STH infection in this region.

Appendix Additional information about parasitic disease surveillance, Mississippi, USA.
