# Supplementary material for: Parasitic Disease Surveillance, Mississippi, USA
Source: Emerg Infect Dis. 2021 Aug;27(8):2201–4. doi: 10.3201/eid2708.204318 (PMC8314824; doi:10.3201/eid2708.204318)
Supplement: Appendix — Additional information about parasitic disease surveillance, Mississippi, USA. [file 20-4318-Techapp-s1.pdf]

Article DOI: https://doi.org/10.3201/eid2708.204318

Parasitic Disease Surveillance, Mississippi, USA

Appendix

Materials and Methods

For *Toxocara* spp. and *S. stercoralis* helminth antibody detection, sera were exposed to antigen-coupled beads, using either recombinant *Toxocara canis* C-type lectin 1 (rTc-CTL-1) antigen for detection of antibodies to *Toxocara* spp (*1*). or a recombinant 31 kDA third stage *S. stercoralis* larval antigen (rSs-NIE-1) (*2*). For intestinal protozoa, two *Cryptosporidium parvum* immunodominant antigens (Cp17 and Cp23) and the *G. duodenalis* variant surface protein 3 (VSP3) (*4*) were employed. Serum was diluted 1:400 in Buffer B (1x PBS, 0.5% polyvinyl alcohol, 0.8% polyvinyl pyrrolidone, 0.5% casein [all Sigma, Burlington MA], 0.3% Tween-20, 0.02% sodium azide) containing 3 µg/mL of *Escherichia coli* extract.

For *Toxocara* spp. and *S stercoralis* antibody testing, the serum/buffer B/*E. coli* extract solution was incubated for 30 minutes, with shaking, at room temperature. For *Cryptosporidium* and *Giardia* antigens, the serum/buffer B/*E. coli* extract solution was incubated overnight at 4°C.

Antigen-specific IgG was detected by incubating specimens in duplicate with magnetic beads and then detecting with 50 ng per well of monoclonal mouse anti-human IgG and 20 ng per well of IgG4 (both Southern Biotech, Birmingham AL) and 250 ng per well of streptavidin-linked R-phycoerythrin reporter (Invitrogen, Waltham MA), as described previously (*4*). Between steps, the magnetic beads were washed three times with 0.05% Tween 20 in PBS, using a BioTek Plate washer (BioTek® Instruments, Winooski, VT). A MAGPIX® reader with xPONENT® software calculated the median fluorescence intensity (MFI) from each bead classification from each well. Background fluorescence from a blank with no serum was subtracted (MFI-bg, reported as MFI).

Samples were considered positive at above 8 MFI for *S. stercoralis* and 23.1 MFI for *Toxocara* spp. A *S. stercoralis* enzyme-linked immunosorbent assay based on crude larval

antigen (CrAg-ELISA) (*4*), with a positive cutoff of 1.7 IU/mL, was used to confirm the results for samples yielding positive results by the initial rSs-NIE-1 MAGPIX® assay. Cutoffs for Cp17 (85 MFI), Cp23 (377 MFI) and VSP3 (84 MFI) were extrapolated from in-house standard curves with cutoffs originally defined by receiver operator characteristic curves as described previously (*3*,*5*) Only samples reacting with both the Cp17 and Cp23 antigens were considered to be positive for prior exposure to *Cryptosporidium*.

References

1. Anderson JP, Rascoe LN, Levert K, Chastain HM, Reed MS, Rivera HN, et al. Development of a luminex bead based assay for diagnosis of toxocariasis using recombinant antigens Tc-CTL-1 and Tc-TES-26. PLoS Negl Trop Dis. 2015;9:e0004168. PubMed https://doi.org/10.1371/journal.pntd.0004168

2. Rascoe LN, Price C, Shin SH, McAuliffe I, Priest JW, Handali S. Development of Ss-NIE-1 recombinant antigen based assays for immunodiagnosis of strongyloidiasis. PLoS Negl Trop Dis. 2015;9:e0003694. PubMed https://doi.org/10.1371/journal.pntd.0003694

3. Priest JW, Moss DM, Visvesvara GS, Jones CC, Li A, Isaac-Renton JL. Multiplex assay detection of immunoglobulin G antibodies that recognize *Giardia intestinalis* and *Cryptosporidium parvum* antigens. Clin Vaccine Immunol. 2010;17:1695–707. PubMed https://doi.org/10.1128/CVI.00160-10

4. Krolewiecki AJ, Ramanathan R, Fink V, McAuliffe I, Cajal SP, Won K, et al. Improved diagnosis of *Strongyloides stercoralis* using recombinant antigen-based serologies in a community-wide study in northern Argentina. Clin Vaccine Immunol. 2010;17:1624–30. PubMed https://doi.org/10.1128/CVI.00259-10

5. Moss DM, Priest JW, Hamlin K, Derado G, Herbein J, Petri WA Jr, et al. Longitudinal evaluation of enteric protozoa in Haitian children by stool exam and multiplex serologic assay. Am J Trop Med Hyg. 2014;90:653–60. PubMed https://doi.org/10.4269/ajtmh.13-0545
